# Functional roles for FEN1 phosphate steering residues in multi-step substrate verification prior to reaction

**DOI:** 10.1016/j.jbc.2026.113225

**Published:** 2026-06-04

**Authors:** Mark J. Thompson, Nur Nazihah B. Md Shahari, Reuben J. Ouanounou, Nathan Gittens, Barbara Ciani, L. David Finger, Jane A. Grasby

**Affiliations:** School of Mathematical and Physical Sciences, University of Sheffield, Sheffield, UK

**Keywords:** DNA–protein interaction, DNA repair, DNA replication, enzyme kinetics, enzyme mechanism, flap endonuclease 1, phosphate steering

## Abstract

Flap endonuclease 1 removes 5′-flaps from double-flap DNA junction intermediates during replication and repair. Substrate recognition and reaction site selection depend on two intrinsically disordered regions: the α4–α5 helical arch, through which the 5′-flap threads prior to catalysis, and the adjacent 3′-flap binding pocket, which allosterically signals disorder-to-order transition of the arch upon sensing a 3′-flap, committing the enzyme–DNA complex towards reaction. The phosphate steering hypothesis proposes that conserved, positively charged amino acids in α4/α5 facilitate passage of 5′-flap DNA through the arch during threading and position the target phosphate diester for hydrolysis; however, supporting evidence is limited and mechanistic details are currently lacking. We investigated functional roles for these residues using kinetic and spectroscopic methods, finding that alanine substitutions of Arg103, Arg104, Arg129 and Lys132 modestly reduce the catalytic rate and the stability of 5′-flap threading. Following arch ordering, distortion of the reacting DNA duplex is necessary for active site transfer of the target cut site, and we identified key roles in this process for two substrate-facing residues from α5, Lys125 and Arg129. Concurrently, ‘back-of-arch’ residues Arg104 and Lys132 contact the +1 phosphate to precisely position the target phosphodiester for hydrolysis. Helicity-disrupting mutations in α4/α5, designed to impair ordering, reduced the catalytic rate and severely inhibited allosteric signaling of 3′-flap recognition to the active site. These findings define critical functional roles for phosphate steering residues in the flap endonuclease 1 mechanism, and inform a deeper understanding of how coordinated substrate verification optimizes targeting specificity to preserve genome integrity.

As an essential component of the DNA replication and repair apparatus, flap endonuclease 1 (FEN1) catalyzes the removal of single-stranded DNA (ssDNA) overhangs, known as 5′-flaps, from DNA junction structures produced during strand displacement synthesis (Fig. 1*A*). It is described as a structure-sensing nuclease due to recognizing target substrates independent of nucleotide sequence, requiring the two DNA helices of the substrate to bend by approximately 100° to create most of the protein–DNA interface (1). This feature ensures selection of DNA junction structures over continuous genomic double-stranded DNA (dsDNA), which must not be indiscriminately hydrolyzed (Fig. 1*B*).

**Figure 1:**
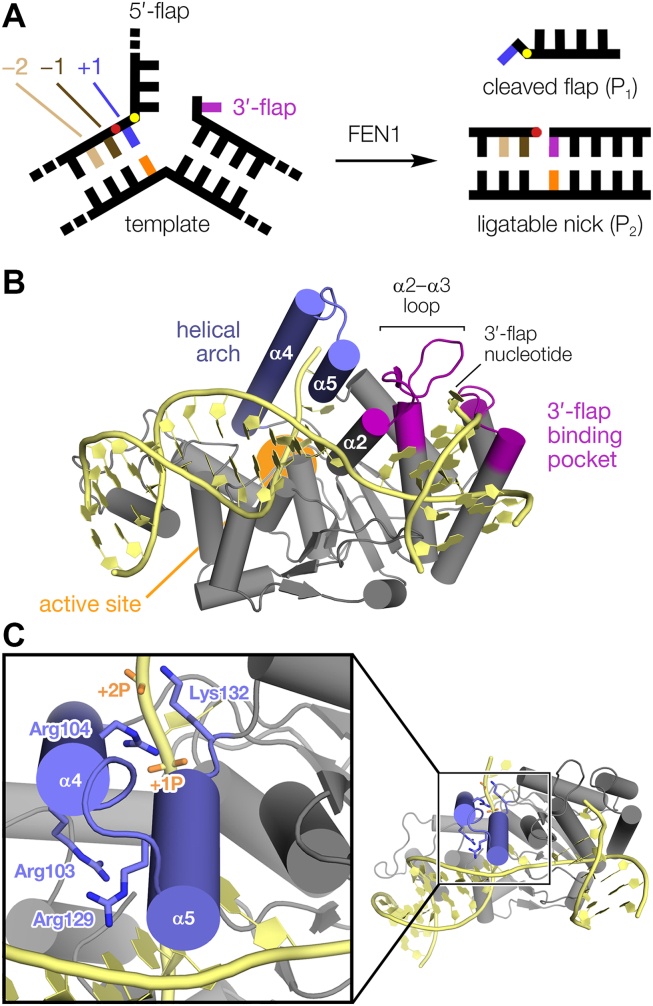
**FEN1 function, structure and phosphate steering residues, and their relation to mechanism.***A*, FEN1 processes the products of strand displacement synthesis arising from DNA replication and repair into a nicked dsDNA structure (P_2_) that can be directly ligated, releasing the 5′-flap as an single-stranded DNA by-product (P_1_). The *red dot* indicates the scissile phosphate diester (*i.e.*, the site of hydrolytic reaction in the substrate), while the *yellow dot* denotes the +1 phosphate. Neighboring nucleobases (−2, −1 and + 1) are numbered relative to the scissile phosphate. *B*, structure of hFEN1-D86N in complex with substrate DNA (PDB code 5UM9) (5), with key protein features annotated and showing the 5′-flap threaded through the ordered helical arch (α4–α5). In this structure, the substrate is positioned to react except that the D86N mutation precludes binding of the second active site metal ion that would activate the attacking water molecule. *C*, top-down view of the same complex, highlighting the positions of phosphate steering side chains (front-of-arch residues Arg103 and Arg129, and back-of-arch residues Arg104 and Lys132; *blue sticks*), as well as the +1 and + 2 phosphate non-bridging oxygen atoms (*orange*).

Early work established the optimal FEN1 substrate as a double-flap conformer of the two-way junction (as shown in Fig. 1*A*), with a single nucleotide 3′-flap and any length of 5′-flap, including zero (2, 3). The hydrolytic reaction occurs one nucleotide into the reacting duplex, which when combined with the requirement for a single nucleotide 3′-flap ensures that FEN1 action produces nicked dsDNA suitable for direct ligation. This high specificity ensures efficient and accurate completion of DNA replication or repair, guarding against genomic instability (4).

Whereas the ideal FEN1 substrate has long been known, the molecular basis for this selectivity was elucidated only relatively recently. Crystal structures of human FEN1 (hFEN1) and paralogues in complex with DNA provided a wealth of information to support more detailed mechanistic understanding (Fig. 1, *B* and *C*) (1, 5). The 5′- and 3′-flap binding sites were clearly defined, and ordering of the α4–α5 flexible loop region was seen in complexes with DNA, to form a structural feature referred to as the ‘helical arch’ that straddles the enzyme’s active site (Fig. 1*B*). Through NMR studies, we previously confirmed earlier assumptions that this region is an unstructured flexible loop in substrate-free FEN1, and while parts of α4 were seen to transiently sample α-helical space, it was concluded that α5 is always disordered (6). Similarly, in X-ray crystal structures of hFEN1 (without DNA substrate), α4 is disordered or partially ordered but α5 is always disordered (7), consistent with the NMR data. In the presence of DNA substrate (or product), published structures indicate a fully ordered helical arch with the 5′-flap (if present) threaded through this protein structural feature (1, 5).

Accordingly, biochemical experiments supported a ‘disorder–thread–order’ model of substrate binding wherein the 5′-flap threads through the initially disordered flexible loop, followed by ordering of α4–α5 to initiate catalysis (8). This requirement for threading means the substrate must have a free 5′-terminus, preventing aberrant processing of continuous ssDNA–dsDNA junctions that arise during normal replication.

A key step in control of FEN1 catalysis was recognized as transfer of the scissile phosphodiester bond onto active site metal ions, or ‘active site transfer’ (9, 10), which was assumed to involve conformational changes in the substrate after initial protein–DNA binding (5, 6, 7). This was seemingly confirmed through a complex between the catalytically impaired hFEN1-D86N mutant and a double-flap substrate, positioned for reaction, where significant deviation of the reacting duplex region from a regular B-DNA structure was observed (5). As implicated in prior functional studies (10), recognition of the +1 phosphate (yellow dot in Fig. 1*A*) seemed to play a particularly important role in active site transfer.

It was also suggested that several positively charged and conserved protein side chains in the helical arch—near to but distinct from the FEN1 active site—help ‘steer’ the substrate into position for reaction in a twisting or rolling type of motion, corresponding to the aforementioned DNA conformational change. These residues (Arg103, Arg104, Arg129 and Lys132; Fig. 1*C*) were termed ‘phosphate steering’ residues, and in addition to a role in moving the scissile phosphodiester to within catalytic distance of the active site metal ions (*i.e.*, active site transfer), they were proposed to help orient the 5′-flap away from the active site during threading to preserve the integrity of flap ssDNA. Thus, phosphate steering was defined as “electrostatic interactions that can dynamically position the phosphodiester backbone” (5).

Distinguishing between these roles in 5′-flap threading and active site transfer is crucial because the helical arch domain must be disordered during the former process but properly structured to enable the latter, since ordering controls assembly of the active site by bringing two essential conserved catalytic residues, Lys93 and Arg100, into position for reaction. This is explained by the disorder–thread–order model that implies threading and active site transfer are distinct processes. Subsequent work offered strong evidence that the key event driving disorder-to-order transition of the arch is recognition of the 3′-flap, which is bound over 20 Å from the active site in a mechanism that effectively makes it an allosteric regulator of active site transfer and, by implication, of catalysis (7).

Despite the intuitive appeal of the phosphate steering hypothesis, and a supportive molecular dynamics study (11), a direct role for the proposed steering residues in either 5′-flap threading or active site transfer has not been conclusively demonstrated. In this paper, we aim to answer important outstanding questions regarding the specific functional role(s) of phosphate steering residues, considering how and where these roles integrate with known molecular details of the FEN1 reaction pathway.

## Results

### Phosphate steering mutants show reduced threading stability

**Figure 2:**
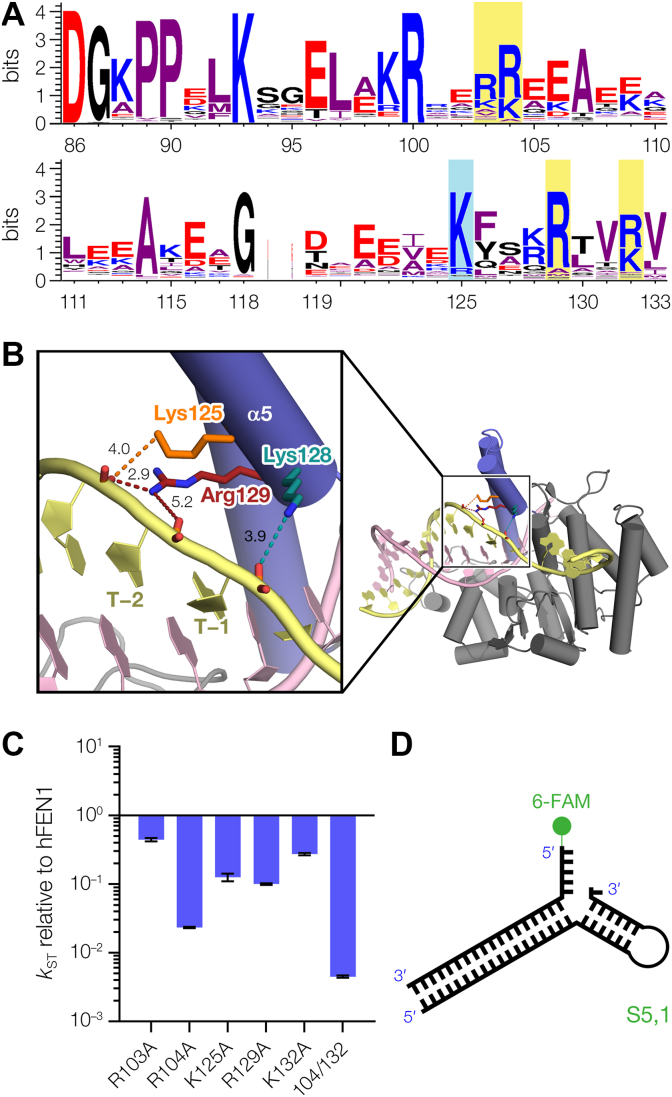
**Phosphate steering contacts are conserved in FEN1s and contribute to reactivity.***A*, sequence logos based on alignment of 179 archaeal and eukaryotic FEN1s illustrate varying degrees of conservation of phosphate steering residues (*yellow highlights*), as noted previously (5). Strong conservation of hFEN1-Lys125 (*cyan highlight*) is also observed. Residue numbering corresponds to the hFEN1 sequence. *B*, three positively charged side chains from α5 make electrostatic contacts to the continuous template strand (*yellow*) of the DNA substrate. Conserved residues Lys125 (*orange*) and Arg129 (*red*) are in close proximity for electrostatic interactions, as is semi-conserved residue Lys128 (*teal*). Distances are labeled in Å and the 5′-flap strand is shown in *pink*. Image prepared using PDB code 5UM9 (5). *C* and *D*, mutation of phosphate steering residues and Lys125 to alanine has a varying impact on FEN1 ST activity (*C*) measured using an idealized double-flap substrate S1 (*D*), noting 104/132 indicates the back-of-arch R104A/K132A double mutant. For R103A, K125A and R129A, rates are reported as mean and standard error from global fitting of time course data *via* nonlinear regression (Fig. S2); replicates were as follows: R103A, n = 4 from two independent experiments performed in duplicate; K125A, n = 3 technical replicates; R129A, n = 4 from two independent experiments performed in duplicate. Results for wt hFEN1, R104A, K132A and 104/132 were obtained similarly and are reproduced from a previous report (5). ST, single turnover.

As noted above, the hFEN1 phosphate steering residues were defined as Arg103 and Arg104 in helix α4, and Arg129 and Lys132 in α5 (Fig. 1*C*). Conservation of positive charge at these positions was seen widely among archaea and eukaryotes (5). Further sequence analysis (Fig. 2*A*), using a set of 179 curated FEN1 sequences reported previously (7), confirmed this earlier conclusion but also revealed strong conservation of Lys125, which contacts the template strand of the substrate along with other α5 residues (Lys128 and Arg129; Fig. 2*B*). We therefore prepared alanine mutants of all five residues in the present study.

These mutations noticeably impacted the reactivity of hFEN1 towards a model double-flap substrate, albeit to different degrees (Fig. 2*C*). Specifically, single turnover (ST) kinetics experiments were performed using an idealized static double-flap substrate S1 that exists in a single conformer of the DNA junction, and contains a 5 nucleotide 5′-flap and the canonical single nucleotide 3′-flap (see Fig. 2*D* for a schematic, and Table S1 and Fig. S1 for sequence and structural information). To elucidate the maximal ST rate (*k*_STmax_), which directly reflects the enzyme’s ability to perform its hydrolytic reaction, substrate (5 nM) and enzyme (400 nM) concentrations were respectively set at approximately an order of magnitude below and above the *K*_M_ value of hFEN1 (∼35 nM) (3, 7, 12) to ensure measured values reflected the maximal ST rate under saturating conditions. Our previous work has demonstrated very similar rates at 400 nM and 1000 nM [E] for hFEN1 and a range of phosphate steering and 3′-flap binding mutants (5, 7), establishing that saturating conditions are adequately achieved at 400 nM enzyme. A 6-carboxyfluorescein label was attached to the 5′-flap terminus to enable quantitative analysis of quenched reaction aliquots by HPLC, as described previously (5, 7). Earlier results indicate that tethering this fluorophore does not impair threading (7, 8), consistent with a report that hFEN1 accommodates 5′-flap lengths of up to 30 nucleotides with no significant impact on ST rate (13).

A very modest impact on *k*_STmax_ was seen for R103A and K132A (within fivefold of wt hFEN1, consistent with previous results for K132A) (5), whereas R129A and K125A were both approximately 10-fold slower (Figs. 2*C* and S2). The other mutant, R104A, exhibited a more substantial rate reduction of 40-fold and an additive effect when combined with K132A, as documented in the original phosphate steering study (5). Although ST rate defects were already known for most of these mutations, their impact on 5′-flap threading has not yet been evaluated.

To address this, we utilized an established assay to compare the proportion of enzyme–substrate complex where the substrate is bound in a threaded state at equilibrium (7, 8). This is achieved by incorporating an additional biotin label at the 5′-flap terminus (substrate S2; see Table S1 and Fig. S1), and exploiting the fact that a streptavidin–biotin complex is far too large to fit through the hFEN1 helical arch, even in a disordered state. Given that threading requires the presence of divalent metal ions, catalytically inert Ca^2+^ is used during equilibration (*i.e.*, enzyme–substrate binding) to prevent reaction. Streptavidin is either added prior to equilibration to prevent threading (‘blocked’ substrate), or introduced after equilibrating the enzyme and substrate to render threading irreversible (‘trapped’ substrate). Single turnover reaction profiles are then followed after introducing excess Mg^2+^ to initiate catalysis.

**Figure 3:**
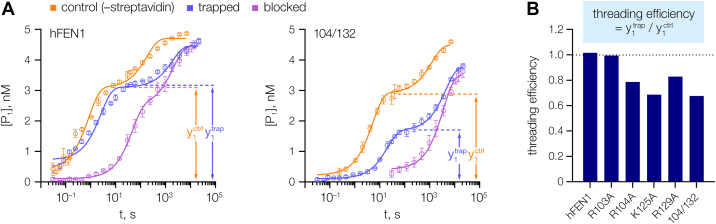
**Phosphate steering mutants exhibit 5′-flap threading defects.***A*, kinetic profiles from threading assays with wt hFEN1 (*left*) and double phosphate steering mutant 104/132 (*right*), using biotinylated substrate S2. With the wt enzyme, trapped substrate decays to the same initial endpoint (defined by the modeled first phase from two-phase exponential curve fitting) as the free substrate with no streptavidin added, implying that the 5′-flap is fully threaded after pre-equilibration of enzyme and substrate. With the double mutant these reactions reach different endpoints, indicating some unbound/unthreaded substrate at the point of introducing streptavidin. Error bars denote SD of n = 4 replicates per condition from two independent experiments performed in duplicate. *B*, threading efficiencies, calculated based on the results in (*A*) and analogous experiments with R103A, R104A, K125A and R129A (Fig. S3). Except for R103A, all mutants exhibit a threading defect. Graphed values represent the ratio of endpoint values for the first reaction phase from global two-phase model fitting.

For wt hFEN1, all of the substrate is known to be threaded when bound to the enzyme (7), revealed by the fact that the initial (‘fast’) phase of the reaction reaches equivalent endpoints for either the trapped or control (no streptavidin) complexes (*i.e.*, y_1_^trap^ = y_1_^ctrl^, as defined and shown in Fig. 3*A*). The blocked substrate reacts 100-fold slower. With FEN1 mutants or substrates lacking a 3′-flap, threading defects are often observed (*i.e.*, y_1_^trap^ < y_1_^ctrl^), as seen for phosphate steering mutants R104A, R125A and R129A, which all exhibited modest but discernible threading deficiencies (Figs. 3*B* and S3). The double mutant R104A/K132A showed a more marked compromise in threading ability as might be expected given its 200-fold rate reduction (Fig. 2*C*), further noting that these ‘back-of-arch’ residues both make important contacts to the +1 phosphate.

Since this is an equilibrium assay and *k*_on_ is diffusion-limited for hFEN1 (14), we have previously interpreted threading deficiencies as representing reduced population and/or lifetime of the threaded (and ordered) complex, which is in turn assumed to be stabilized by the additional protein–DNA contacts formed upon helical arch structuring (6, 7). In an earlier report, based on multiple lines of evidence from kinetic and spectroscopic experiments, it was concluded that helical arch ordering is a key step in the FEN1 reaction pathway that ‘locks’ the 5′-flap in position, stabilizing the complex to be longer-lived and proceed towards active site transfer and catalysis (7); otherwise, the FEN1–DNA complex is more prone to dissociation. The data implied that ordering of the helical arch is a strict requirement for the FEN1 reaction and is controlled by 3′-flap binding. If phosphate steering mutations do not affect this disorder-to-order transition, the observed threading deficiencies likely reflect a destabilizing influence on the FEN1–DNA complex (*i.e.*, increased *k*_off_). As such, it was unsurprising to observe a meaningful threading defect for R104A and R104A/K132A (hereafter 104/132) given these residues make contacts with the +1 phosphate group that sits directly beneath the helical arch in the reactive configuration (Fig. 1*C*). Certain observations suggest the threading and rate defects for 104/132 may reflect a decreased binding lifetime: the *K*_d_ of wt hFEN1 has been reported as 3.3 to 4.8 nM (depending on 5′-flap length from 6–50 nucleotides) (15), based on single-molecule FRET experiments, whereas we previously determined the *K*_d_ of 104/132 as 44.9 nM *via* bulk FRET measurements (with a 5′-flap length of 6 nucleotides) (5). Assuming *k*_on_ is unaffected, the 200-fold rate deficiency for 104/132 is then at least partly explained by an order of magnitude increase in *k*_off_ (arising from the loss of +1 phosphate contacts), yet there also seems to be a role for functional defects in the threaded-and-ordered FEN1–DNA complex, prompting us to consider potential deficiencies in active site transfer as well.

Interestingly and somewhat unexpectedly, the trend in threading defects differed to that seen in kinetics results, with the K125A and R129A mutants showing greater threading deficiency than initially anticipated based on ST rates alone (10-fold slower than wt hFEN1). This suggested that α5 contacts to the continuous DNA template strand, and not just the 5′-flap, might be more important in the FEN1 mechanism than has previously been recognized, prompting further investigation of their role in active site transfer, alongside Arg104 and Lys132.

### Resolving active site transfer measurements with FEN1 substrates

The phosphate steering hypothesis proposed that as well as promoting threading, certain positively charged protein side chains play a role in active site transfer (*i.e.*, guiding the reacting DNA duplex into the FEN1 active site), with the above results also suggesting a role for Lys125. We considered how best to interrogate this process in the present context, noting that previous efforts to measure active site transfer, by ourselves and others, have exploited modified substrates incorporating 2-aminopurine (2AP) nucleobases near to the reaction site.

Since 2AP fluorescence is strongly quenched by neighboring nucleobases in the absence of enzyme (16), measurements of 2AP fluorescence have been used to infer active site transfer. One such approach (17) relied on static double-flap substrate S3, incorporating adjacent 2AP bases at the +1 and −1 positions (see Table S1 and Fig. S1 for sequence and structural information). A threefold increase in fluorescence was reported over the first 100 s of a Mg^2+^-supported reaction in the presence of slow-reacting hFEN1 mutant D34A. No product formation was detected over this timescale, so the fluorescence change was interpreted as observation of active site transfer prior to catalysis (17). Since this result contradicted our previous findings, we explored 2AP fluorescence changes further by investigating S3 with wt hFEN1 and a similar mutant, D34N, which exhibits ∼80000-fold slower reaction with substrate S1 (*k*_STmax_ = 0.013 vs 1020 min^−1^) (7) and evidence for off-target hydrolysis (Fig. S4).

We have previously demonstrated that addition of catalytically inert Ca^2+^ ions to hFEN1 complexes with 2AP-containing substrates produces a threaded enzyme–substrate complex and alters the time-resolved 2AP fluorescence parameters of the substrate (9, 18). In our hands, there was no effect of Ca^2+^ on steady-state fluorescence of the wt hFEN1–S3 complex over the first 100 s (Fig. S5*A*, cyan plot), whereas addition of Mg^2+^ ions produced a rapid and significant increase in steady-state fluorescence (Fig. S5*A*, purple plot), in agreement with the aforementioned report (17) and readily attributable to the disruption of quenching interactions upon hydrolytic reaction. Under these conditions, the wt hFEN1 reaction is rapid (half-time ∼40 ms) and is essentially complete at the first data point (5 s), producing an approximately 10-fold increase in fluorescence. A slower reaction phase (assumed to reflect unbinding/rebinding of unproductively bound substrate, in line with previous studies) (3, 7) explains the additional increase seen from 5 to 100 s. Thus, fluorescence changes for wt hFEN1–S3 are fully explicable in terms of substrate hydrolysis, without invoking a contribution from active site transfer.

We proceeded to study the D34N–S3 complex. As with wt hFEN1, no fluorescence changes were observed in the presence of Ca^2+^ ions over 100 s (Fig. S5, beige plots). In this case, Mg^2+^ also failed to induce a change on the same timescale (Fig. S5, red plots), consistent with the essentially undetectable product formation over this period with S1 (Fig. S4). However, we did observe a gradual increase in fluorescence over 4 h (data not shown), after which time the slow hydrolysis of S1 by hFEN1-D34N is mostly complete (half-time = 55 min). We were therefore unable to detect possible active site transfer *via* 2AP fluorescence increases during the early stages of mutant FEN1 reactions, and steady-state fluorescence changes with wt enzyme were attributable solely to product formation.

**Figure 4:**
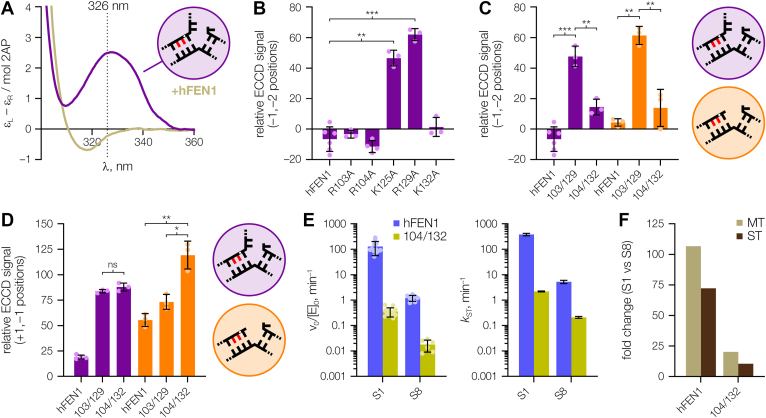
**Phosphate steering residues play distinct roles in the FEN1 reaction mechanism.***A*, representative ECCD spectra of substrate S4 alone (*purple plot*) or after mixing with hFEN1 (*beige plot*). The initial maximum at 326 nm, due to exciton coupling between adjacent 2APs (20), is reduced to near-zero in the presence of enzyme, indicating a significant conformational change of the DNA upon binding (interpreted as active site transfer). A schematic of S4 is shown in the *purple circle*, with tandem 2AP residues at the −1 and −2 positions highlighted in red. *B*, among the phosphate steering single mutants, K125A and R129A are severely compromised in their ability to bring about active site transfer with S4. Signal intensities at 326 nm are expressed as a percentage of that for substrate alone on the same experimental run. Statistical analysis *via* pairwise two-tailed, two-sample, unequal variance Student’s t-tests revealed significant differences *versus* wt hFEN1 for K125A (*p* = 0.0015) and R129A (*p* = 0.0002). *C*, ECCD results for front-of-arch mutant 103/129 and back-of-arch mutant 104/132, with S4 (*purple bars*) and related exonucleolytic substrate S5 (*orange bars*; see *orange circle* for schematic). Statistical analysis as above showed significantly compromised active site transfer for 103/129 *versus* 104/132 with both S4 (*p* = 0.0031) and S5 (*p* = 0.0098); 103/129 also exhibited impaired active site transfer compared with hFEN1 (S4, *p* = 0.0001; S5, *p* = 0.0012). *D*, parallel results to (*C*) with analogous substrates S6 (*purple bars and circle*) and S7 (*orange bars and circle*), containing 2AP residues at the +1 and −1 positions (indicated in *red*). In these substrates, reduced ECCD signal intensity with wt hFEN1 is interpreted as proper active site positioning of the substrate for reaction. A difference between mutants is seen with S7, where the +1 phosphate is the 5′-terminus of the reacting strand, and statistical analysis, again as defined above, demonstrated significantly impaired substrate positioning for 104/132 *versus* 103/129 (*p* = 0.0138) and wt hFEN1 (*p* = 0.0065). With S6, no significant difference between the two mutants was observed (*p* = 0.1961). *E*, rates of reaction under MT (*left*) or ST (*right*) conditions for model double-flap substrate S1 and S8, containing a methylphosphonate substitution at the +1 position. Both wt hFEN1 (*blue bars*) and 104/132 (*olive bars*) show reduced reactivity with S8. *F*, ratio of average rates for S1/S8 under MT (*beige bars*) and ST (*brown bars*) conditions shows a much lower impact of +1 methylphosphonate substitution for the back-of-arch mutant 104/132, supporting the proposal that Arg104 and Lys132 interact with the +1 phosphate. Note: Graphs in (*B–D*) include overlaid scatter plots of replicate ECCD measurements, which were each performed independently on different experimental runs (*B*, n = 6 for hFEN1, n = 4 for R104A, n = 3 otherwise; *C*, n = 6 for hFEN1, n = 3 otherwise; *D*, n = 3 for all conditions); in panel (*E*), MT, n = 12 for hFEN1 with both substrates, n = 8 for S1 with 104/132, and n = 6 for S8 with 104/132; in all cases, *error bars* denote SD. ST data in (*E*) were derived *via* global model fitting to kinetic data, as with previous examples, for reactions performed in triplicate (Fig. S6). MT, multiple turnover; ST, single turnover; ECCD, exciton-coupled CD.

In contrast, DNA conformational changes corresponding to active site transfer can be observed spectroscopically in near-UV CD spectra using the exciton-coupled CD (ECCD) assay, which similarly relies on substrates with tandem 2AP pairs (7, 9, 10, 12, 18, 19). Neighboring (*i.e.*, stacked) 2AP bases in dsDNA form an exciton pair, whose CD signal intensity maximum at 326 nm is highly sensitive to the relative orientation of the two bases (17, 20). When 2AP residues are placed at the −1 and −2 positions of an idealized double-flap substrate such as S4 (Table S1 and Fig. S1), the initial ECCD signal in the presence of Ca^2+^ ions is reduced to near-zero upon addition of wt hFEN1 (Fig. 4*A*). This spectral shift is assumed to reflect significant conformational change in the bound substrate, namely active site transfer, based on prior functional and crystallographic observations (5, 9, 10).

### Phosphate steering residues have distinct roles in active site transfer

The ECCD assay was applied to interrogate the role of phosphate steering residues in active site transfer. Among the present set of point mutants, only K125A and R129A showed defects in this conformational change with double-flap substrate S4 (Fig. 4*B*), suggesting a role for α5 contacts in promoting the FEN1 reaction. Stated differently, conserved electrostatic contacts made from α5 of hFEN1 to the template strand of the substrate may be important in attaining and/or stabilizing the catalytically competent conformation.

Mutation of ‘front-of-arch’ phosphate steering interactions (R103A/R129A, hereafter 103/129) or the corresponding back-of-arch contacts (104/132; see Fig. 1*C*) also allowed a role in active site transfer to be discerned for the former, by utilizing both S4 and an analogous exonucleolytic substrate (S5, where the 5′-flap length is zero; see Table S1 and Fig. S1 for sequence and structural information). Only the 103/129 double mutant displayed significantly compromised active site transfer with these substrates (Fig. 4*C*), indicating an important role in active site transfer for Arg103 and Arg129, but not Arg104 and Lys132. Together with the data for K125A, these results suggest that front-of-arch contacts from α4 and α5 can promote active site transfer, possibly in concert with short-range ‘rocking’ motions of the structured arch that were proposed based on NMR and single-molecule FRET data (6), and may help ‘roll’ the reacting duplex region into the enzyme active site. Other results with a hyperthermophilic FEN1 (from *Archaeoglobus fulgidus*) showed that threading efficiency and active site transfer ability were strongly temperature-dependent, improving as the temperature was raised and the degree of disorder in the arch region of the substrate-free protein increased (7).

Intriguingly, the above findings (Figs. 3*B* and 4*C*) indicate that while the back-of-arch residues (Arg104 and Lys132) contribute towards threading stability, presumably by contacting the critical +1 phosphate, they make little (if any) contribution to active site transfer. Their role was further probed using substrates S6 and S7, which are analogous to S4 and S5 but incorporate 2AP modifications at the +1 and −1 positions (as opposed to the −1 and −2 positions; see Table S1 and Fig. S1 for details). In earlier work we concluded that spectral changes with +1/−1 modified substrates provide a readout of proper substrate positioning for catalysis in the FEN1 active site, distinct from active site transfer assessed by substrates with −1/−2 modifications (7, 10). When interrogated with substrate S6 in the ECCD assay, both phosphate steering double mutants (103/129 and 104/132) showed comparable defects in substrate positioning (Fig. 4*D*). However, with exonucleolytic substrate S7 (where the +1 phosphate corresponds to the 5′-terminus of the reacting strand), the 104/132 double mutant was significantly compromised in substrate positioning compared to wt hFEN1, whereas 103/129 was not. These results are consistent with the established role for back-of-arch phosphate steering residues Arg104 and Lys132 in positioning the +1 phosphate to correctly define the site of reaction (5).

An alternative approach to investigate these interactions was to neutralize the negative charge of the +1 phosphate group by methylphosphonate substitution, as in double-flap substrate S8 (see Table S1 and Fig. S1). With wt hFEN1, this substrate was processed around 100-fold slower than unmodified counterpart S1 under both multiple and ST conditions (Fig. 4*E*, blue bars, and Fig. S6*A*). Significantly, the impact on rate was much less for the 104/132 double mutant, with only around a 10-fold rate reduction under the same conditions (Fig. 4*E*, beige bars, and Fig. S6*B*); the ratio of average rates for all four protein/substrate combinations is summarized in Figure 4*F*. A conservative approach was adopted in these experiments, using 1000 nM [E] to accommodate potential weaker binding and ensure saturating conditions were maintained with both substrates, especially considering the slow rates observed with S8 (Fig. S6). These methylphosphonate substitution results reinforce a functional role for electrostatic interactions between back-of-arch phosphate steering side chains (*i.e.*, Arg104 and Lys132) and the substrate +1 phosphate (5), and imply involvement of these contacts in the rate-determining step of the FEN1 reaction. Since earlier work established that this step corresponds to conformational change of protein and/or substrate (10), it seems likely that ST rate defects (Fig. 2*C*) may be explained in terms of compromised active site transfer ability and/or substrate positioning, after threading has occurred.

### Helix α5 is a key mediator of the FEN1 reaction pathway

Taken together, the above findings confirmed a role for phosphate steering residues in the hFEN1 reaction mechanism. However, since mutation of these residues maps to functional impacts on either 5′-flap threading, active site transfer or substrate positioning, all of which are essential for efficient reaction, it becomes apparent that detrimental effects arise through loss of specific contacts. Our results therefore imply that positively charged phosphate steering side chains have discernible functional roles, rather than merely providing a generalized region of positive charge to non-specifically promote threading and/or positioning of substrate DNA.

We have particularly uncovered an important role for contacts made from hFEN1 to the template strand *via* Lys125 and Arg129, both situated in helix α5 (Fig. 2*B*). The functional significance of these contacts has not been fully appreciated to date, despite the fact they show strong conservation at the residue level across archaea and eukaryotes (Fig. 2*A*). Earlier evidence for their role is, in fact, apparent from results obtained with hFEN1 mutant L130P, for which structuring of α5 was presumed to be either inhibited or prevented completely (18). With an idealized double-flap substrate, this mutant reacted 3000-fold slower than wt hFEN1 under ST conditions, and significantly, the ECCD assay revealed severely impaired active site transfer ability.

**Figure 5:**
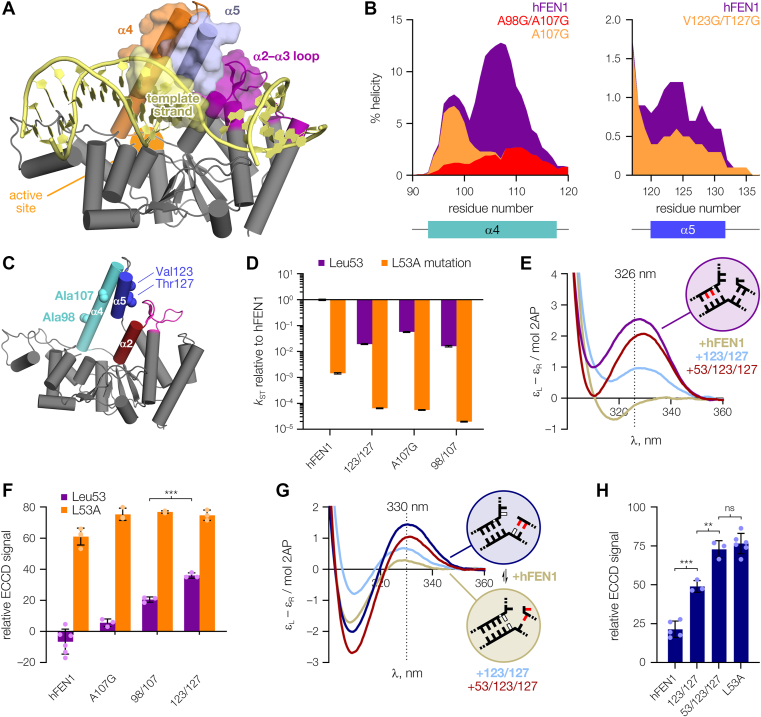
**Arch ordering is important in the FEN1 mechanism and relies upon 3′-flap interaction.***A*, partial surface renders (*transparency*) emphasize the multiple interactions made by helix α5 once ordered, based on PDB structure 5UM9 (5). *B*, AGADIR predictions (at 20 °C) of α-helicity for α4 (*left*) and α5 (*right*) sequences, in wt hFEN1 (*purple*) or with the indicated mutations. *C*, location of residues mutated to glycine in the hFEN1 structure. *D*, ST reaction rates with S1 for the indicated proteins (x-axis), without (*purple bars*) or with (*orange bars*) an additional L53A wedge residue mutation. *E*, representative ECCD spectra for S4 without (*purple plot*) or with (*beige plot*) addition of hFEN1, superimposing results for α5 mutant V123G/T127G (123/127; *cyan plot*) and triple mutant L53A/V123G/T127G (53/123/127; *red plot*), where active site transfer is essentially abolished. *F*, ECCD results for proteins in (*D*) with S4. Note that mutating α5 (123/127) affects active site transfer more than mutation of α4 in A107G and A98G/A107G (98/107). Statistical analysis *via* a pairwise two-tailed, two-sample, unequal variance Student’s *t* test revealed active site transfer is significantly worse for 123/127 *versus* 98/107 (*p* = 0.0004). Combination mutants with L53A behaved similarly to L53A alone, displaying severely impaired active site transfer. *G*, representative ECCD spectra for S9, which exists mostly in single-flap form when alone in solution (*blue plot* and schematic in *blue circle*, with 2AP bases in *red*), but displays a much-reduced signal in the presence of wt hFEN1 (*beige plot*), consistent with conformational shift to the double-flap form with a single nucleotide 3′-flap (schematic in *beige circle*). This ability to induce a 3′-flap is partially inhibited in α5 mutant 123/127 (*cyan plot*), and substantially impaired for combination wedge mutant 53/123/127 (*red plot*). *H*, ECCD results for S9 with the specified proteins (x-axis). Statistical analysis as above demonstrated significantly compromised 3′-flap formation by 123/127 *versus* wt hFEN1 (*p* = 0.0002), and also for combination mutant 53/123/127 *versus* 123/127 (*p* = 0.0013). As for S4, the combination mutant acts similarly to L53A alone (*p* = 0.4694). Note: Panel (*D*) reports ST rates and standard error from global model fitting to kinetic data, as in previous cases, for replicate reactions (Fig. S2); replicates were as follows: 123/127, n = 4 (using rapid quench flow technique, QF) from two independent experiments performed in duplicate plus n = 3 technical replicates (manual sampling for longer time points); 53/123/127, n = 6 from two independent experiments performed in triplicate (manual sampling); A107G, n = 4 (QF) from two independent experiments performed in duplicate plus n = 3 technical replicates (manual sampling for longer time points); 53/107, n = 3 technical replicates (manual sampling); 98/107, n = 4 (QF) from two independent experiments performed in duplicate plus n = 3 technical replicates (manual sampling for longer time points); 53/98/107, n = 6 from two independent experiments performed in triplicate (manual sampling). Previously reported values are used for wt hFEN1 and L53A (5, 7). Graphs in (*F* and *H*) include overlaid scatter plots of replicate ECCD measurements, which were each performed independently on different experimental runs (*F*, n = 4 for hFEN1/Leu53 and n = 3 otherwise; *H*, n = 5 for hFEN1, n = 6 for L53A, n = 3 otherwise); *error bars* denote SD. ST, single turnover; ECCD, exciton-coupled CD.

Although α5 is disordered in the absence of substrate (6), the multiple contacts it makes once structured (to α4, the α2–α3 loop region and the substrate template strand; Fig. 5*A*) position it as a key structural feature in the allosteric model of FEN1 function that links 3′-flap recognition to catalysis (1, 7, 14). In this model, ‘sensing’ of a 3′-flap by the α2–α3 loop (over 20 Å from the active site) initiates the essential disorder-to-order transition of the helical arch (α4 and α5), leading to active site transfer and catalysis. Consistent with the disorder–thread–order model of FEN1 function, these events all appear to occur after threading, but α5 has strikingly low α-helical propensity in the free protein based on predictions from the AGADIR algorithm (21) (Fig. 5*B*). Nevertheless, the present results indicate that not only must α5 order properly to enable the aforementioned processes, specific contacts with the substrate are necessary for efficient reaction. We hence proceeded to investigate the role of 3′-flap recognition in helical arch ordering.

### Ordering of α4 and α5 is driven by 3′-flap recognition

To pursue further evidence for disorder-to-order transition of the helical arch and its dependence upon 3′-flap recognition, glycine mutations were introduced in α4 and α5 at sites predicted to substantially disrupt helix formation according to AGADIR (Fig. 5, *B* and *C*). The α4 sequence contains two distinct maxima in helical character such that two variants were selected for analysis: the A107G mutation was expected to sharply inhibit structuring of the top half of α4 (Fig. 5*B*), whereas the double mutation A98G/A107G was predicted to impair structuring of the full sequence. For α5, a double mutation (V123G/T127G) was found necessary to appreciably reduce its low inherent helical propensity.

Kinetic evaluation of these new mutants, again under ST conditions with substrate S1, showed a 25-fold rate reduction for hFEN1-A107G and 100-fold slower reaction for the two double mutants, relative to wt hFEN1 (Fig. 5*D*, purple bars, and Fig. S2). To maintain saturating conditions, these reactions with performed at 1000 nM [E] as a precautionary measure due to the substantial impact of the mutation(s) on reaction rate. While the observed kinetic effects are consistent with impaired arch ordering in the glycine mutants, it is apparently not prevented completely because *k*_STmax_ remains appreciably faster than seen with the analogous substrate lacking a 3′-flap, which reacts almost 1000-fold slower (7). Hence, the next question to ask was how mutation of the 3′-flap interaction would impact activity of the glycine mutants. Recognition of the 3′-flap was shown to rely principally upon a conserved ‘wedge residue’ in the middle of the α2–α3 loop region, corresponding to Leu53 in hFEN1 (7); mutating this residue to alanine slowed the ST reaction with S1 by a factor of ∼1000, similar to removal of the 3′-flap. In the same study, characterization of hFEN1-L53A and other 3′-flap binding variants indicated a statistically significant correlation between ST rate and active site transfer ability (measured *via* the ECCD assay with substrate S4), suggesting 3′-flap recognition as the key event that initiates arch ordering.

Each of the three glycine mutants was therefore prepared with an additional L53A mutation, then ST rates were measured (again at 1000 nM [E]) to assess the role of 3′-flap binding in arch ordering (Fig. 5*D*, orange bars). The observed activity of these combination mutants was drastically reduced in every case, to a similar extent to that seen for hFEN1-L53A. Overall, the kinetic results with α4–α5 glycine mutants seem consistent with the proposal that the arch must order fully to promote reaction, and that this ordering is critically dependent upon wedge residue Leu53 binding a 3′-flap.

Interestingly, the ECCD assay with S4 (Fig. 5, *E* and *F*) revealed a greater impact on active site transfer for mutation of α5 (V123G/T127G) compared with α4 (A98G/A107G; Fig. 5*F*, purple bars), despite the fact α5 is more distant from the active site, and that as noted earlier, two invariant catalytic residues (Lys93 and Arg100) lie at the base of α4 and rely upon its ordering to correctly position them for reaction (18). Meanwhile, the L53A combination mutants (Fig. 5*F*, orange bars) gave essentially identical ECCD results and were similarly compromised to the hFEN1-L53A single mutant, suggesting that structuring of α4 may first rely upon that of α5 in a mechanism dependent on the wedge residue.

The α5 glycine mutations also compromised the previously demonstrated ability of hFEN1 to induce a 3′-flap in equilibrating ‘upstream’ substrate S9 (see Table S1 and Fig. S1), which can exist as either a single- or double-flap conformer; earlier work established that the single-flap form predominates in free solution, whereas addition of hFEN1 strongly shifts the equilibrium towards the double-flap conformer, based on the ECCD signal at 330 nm (Fig. 5*G*) (7). Here, the V123G/T127G double mutant showed reduced capacity to induce the 3′-flap conformer, and the combination mutant (L53A/V123G/T127G) proved essentially unable to reconfigure the substrate, similarly to the hFEN1-L53A point mutant (Fig. 5*H*). If structuring of α4 first requires that of α5 as inferred above, the foregoing results are entirely consistent with our earlier assumption that failure to properly order the arch must result in shorter-lived binding of DNA, and that interaction of wedge residue Leu53 with the 3′-flap is a crucial contact in mediating this structural transition that commits the hFEN1–DNA complex towards reaction (7).

## Discussion

The phosphate steering hypothesis proposed that FEN1 exploits a group of conserved or semi-conserved, positively charged residues to dynamically guide the phosphodiester backbone of the reacting DNA strand and thereby control reactivity (5). These residues are thought to orient the 5′-flap DNA away from the active site during threading (as the flap passes through the disordered helical arch), and to play a direct role in positioning the scissile phosphodiester group for reaction once the helical arch has ordered.

This paper offers new evidence supporting defined roles for phosphate steering residues in the substrate conformational changes necessary for FEN1 reaction. Specifically, front-of-arch residues Arg103 and Arg129 promote transfer of the reacting portion of the substrate duplex into the enzyme’s active site, after 5′-flap threading. While active site transfer was originally perceived as local DNA unpairing (9), later findings informed a refined understanding described more generally as distortion or rolling of the reacting duplex to access the active site *via* an irregular DNA structure (5). In this study we identified α5 as a mediator of this substrate motion, including an unexpected role for Lys125 (another conserved front-of-arch residue), suggesting it should be added to the phosphate steering repertoire. Major contributions to the active site transfer process appear to come from both Lys125 and Arg129 (in helix α5), with a potential minor role for Arg103 (in α4), consistent with the level of conservation of each of these residues across eukaryotic and archaeal FEN1s. As mentioned above, our results suggest that front-of-arch contacts, in particular those to the template strand from α5, may be directly involved in a motion involving the ordered helical arch that rolls the reacting duplex into the active site, as also proposed elsewhere (5, 6).

Furthermore, precise positioning of the scissile and +1 phosphate (relative to the active site metal ions) is necessary to achieve the catalytically competent conformation and define the correct site of reaction, in a subtly related but distinct process to active site transfer. Final substrate positioning relies on interactions of the +1 phosphate with back-of-arch phosphate steering residues Arg104 and Lys132, as proposed in crystallographic studies (5) and supported here by multiple lines of evidence from kinetic, spectroscopic (*i.e.*, ECCD) and methylphosphonate substitution experiments.

These established functional roles for phosphate steering residues emphasize how the helical arch actively participates in the FEN1 reaction pathway, rather than merely serving as a passive structural feature as originally thought (*i.e.*, to select for 5′-flaps as opposed to continuous ssDNA) (1, 8, 13). The arch could, in fact, be described as a facilitator of communication due to the number of important contacts made in the catalytically competent conformation. These contacts recognize both strands of the reacting DNA duplex and bridge the 3′-flap (in the upstream duplex) to the active site in what has been characterized as an allosteric process (7).

In the current model, recognition of the 3′-flap initiates an allosteric cascade wherein ordering of the α2–α3 loop (which forms the major contacts in the 3′-flap binding pocket) and then the adjacent helical arch (α5 followed by α4) leads to active site transfer in a fully structured hFEN1–DNA complex. Once the scissile phosphate group is transferred onto the active site metal ions, the hydrolysis reaction will be rapid. On top of the general requirement for FEN1 to bind at a DNA junction, the allosteric cascade mechanism could be thought of as sequentially validating different substrate features, since our results show that a loss of contacts at any stage of this mechanism will compromise the efficiency of the process.

Although 3′-flap contacts are the most influential in this context, phosphate steering interactions with both the template and reacting strands must be present to enable efficient active site transfer and substrate positioning. As such, these residues have important roles in ensuring substrate specificity and defining the site of reaction. The molecular details of the overall allosteric cascade process might therefore be thought of as multi-step substrate verification—providing an elaborate example of kinetic proofreading, which is widely exploited by nucleic acid binding proteins to achieve high specificity (22, 23) and often relies on intrinsically disordered regions (24, 25, 26)—since efficient FEN1 reaction relies not only upon 3′-flap recognition but also requires transmission of this structural information to the active site in a series of conformational changes of both protein and DNA substrate, including disorder-to-order transitions of the α2–α3 loop and helical arch. In turn, these conformational changes depend upon specific, conserved contacts with the substrate from multiple protein residues. The present results illustrate how some of these contacts are provided by phosphate steering residues, affirming their important functional roles in mediating active site transfer and proper substrate positioning for reaction. Although technically challenging, it may be informative to further dissect the relative influence of phosphate steering contacts on binding lifetime and conformational changes leading to reaction, by measuring *k*_off_ for hFEN1 and relevant mutants in future work.

The question of whether phosphate steering residues are involved in 5′-flap threading is harder to resolve, however, since their exact positioning during this process is uncertain due to the disordered state of the arch at this stage (6, 8). Considering that the effects of mutating these residues can be satisfactorily explained in functional terms (with reference to the fully threaded-and-ordered complex), and because shorter ssDNA products arising from reaction in the 5′-flap are not observed with these mutants, it is difficult to support any direct role for these residues in promoting threading itself.

Nevertheless, they may indirectly facilitate threading by influencing the structural dynamics of the disordered helical arch. As is apparent from sequence analysis (Fig. 2*A*), similar numbers of positively and negatively charged residues are distributed across the α4–α5 region. Assessment using the Classification of Intrinsically Disordered Ensemble Regions tool (27) concluded that the charge distribution in this domain serves to maintain its disordered state in an ‘open’ rather than ‘collapsed’ conformation, as would be ideal for threading (6). Positively charged phosphate steering residues must necessarily contribute to this structural characteristic, inferring an indirect but significant effect in maintaining the arch in an open form conducive to 5′-flap threading.

In summary, we conclude that phosphate steering residues do have significant roles in orienting the FEN1 substrate for reaction. However, a complex interplay between these contacts, and the consequences of 3′-flap binding transmitted through conformational changes of the protein and the DNA substrate (including disorder-to-order transitions in the former), is ultimately responsible for the exquisite specificity of the structure-sensing FEN1 nuclease.

## Experimental procedures

### Plasmid constructs

All new hFEN1 mutants were generated from the previously reported pET-28b-hFEN1-(His)_6_ construct (3) by site-directed mutagenesis, using the QuikChange protocol (Agilent Technologies, Inc.) as described (7). Mutagenic primers were purchased from Thermo Fisher Scientific UK (desalted grade), reconstituted in ultrapure water and used as supplied. The sequences were: D34N – 5′-ctcatagaggcattaatggccaccttacggccaaa-3′, 5′-tttggccgtaaggtggccattaatgcctctatgag-3′; A98G – 5′-ctcactgcgtttgcccagctcgcctga-3′, 5′-tcaggcgagctgggcaaacgcagtgag-3′; A107G – 5′-agctgcttctctccctcagcccgcc-3′, 5′-ggcgggctgagggagagaagcagct-3′; V123G/T127G – 5′-caccagccgcttaccgaatttttccccctcctgctcggc-3′, 5′-gccgagcaggagggggaaaaattcggtaagcggctggtg-3′; K125A – 5′-ccagccgcttagtgaatgcttccacctcctgctcgg-3′, 5′-ccgagcaggaggtggaagcattcactaagcggctgg-3′.

### Protein expression

Proteins were expressed in Rosetta (DE3)pLysS competent cells cultured in 2 × YT medium, following reported procedures for cell growth, induction, collection and lysis (7).

### Protein purification

Protein purification was accomplished using an ÄKTA Pure system at 4 °C as previously described (7). Briefly, proteins were purified *via* Ni-NTA immobilized metal affinity chromatography followed by anion exchange and heparin affinity chromatography. Stock solutions were prepared in storage buffer (50 mM HEPES pH 7.5, 100 mM KCl, 1 mM CaCl_2_, 5 mM DTT, 0.02% NaN_3_, 50% v/v glycerol) and kept at −20 °C until needed. Protein-containing fractions were identified and final sample purity was assessed *via* SDS–PAGE.

### Oligonucleotide synthesis and DNA substrates

All oligonucleotides listed in Table S1 were purchased from LGC Biosearch Technologies or Kaneka Eurogentec SA, with HPLC purification. Samples were dissolved in ultrapure water and stock concentrations measured by A_260_ using calculated extinction coefficients. Prior to use, DNA constructs (Table S2 and Fig. S1) were annealed in folding buffer (50 mM HEPES pH 7.5, 100 mM KCl) by heating at 95 °C for 5 min, then allowing samples to stand at room temperature for 30 min.

### Single turnover enzyme kinetics

To determine *k*_STmax_ values, reactions carried out at 37 °C with 400 nM or 1000 nM enzyme (as specified in Figs. S2) and 5 nM substrate S1 were followed by quenching aliquots at appropriate time points, as documented previously (7). For faster reactions, the rapid quench-flow technique was employed using an RQF-63 instrument (TgK Scientific Ltd), in line with prior reports (3, 5, 7, 18). Briefly, solutions of enzyme and substrate were prepared at 2 × final reaction concentration in buffer RB (50 mM HEPES pH 7.5, 100 mM KCl, 8 mM MgCl_2_, 2.5 mM DTT, 0.1 mg mL^–1^ BSA). For each time point, 80 μl aliquots of enzyme and substrate were mixed and quenched in 1.5 M NaOH containing 20 mM EDTA, after a controlled time delay of 0.0045 to 91.041 s. For slower reactions, manual sampling was performed as described with 250 mM EDTA (pH 8.0) used as quenching agent (7).

For all quenched aliquots, the amount of product was quantified *via* denaturing HPLC using a Transgenomic WAVE system equipped with an L-7485 fluorescence detector and a 4.6 × 50 mm OligoSep Cartridge (ADS Biotec, Inc). Product and substrate were separated using previously reported buffers and gradient conditions (7). Specifically, an injected sample with ≥300 fmol DNA was eluted using buffer A (0.1% MeCN, 2.5 mM tetrabutylammonium bromide, 1 mM EDTA) and buffer B (80% MeCN, 2.5 mM tetrabutylammonium bromide, 1 mM EDTA), using the following gradient: 5 to 30% B over 1 min, 30 to 55% B over 4.5 min, 55 to 100% B over 1.6 min, 100% B for 1.4 min, 100–5% B over 0.1 min, 5% B for 2.4 min. Integration of the resultant chromatograms was used to determine the ratio and hence the concentrations of product and substrate with respect to time. If multiple product peaks were observed, the combined total was considered for rate measurements. The first-order rate constant (*k*_STmax_) was derived by global fitting to the experimental data using nonlinear least squares regression in GraphPad Prism 7 (https://www.graphpad.com/) or later as previously described. One- and two-phase exponential models were tested for each dataset, and model selection was informed by Akaike’s information criterion (7).

### Multiple turnover enzyme kinetics

Normalized rates of reaction (*v*/[E]) were determined at 37 °C with 50 nM substrate S1 and enzyme (10 pM hFEN1 or 200 pM R104AK132A), or with 50 nM methylphosphonate-containing substrate S8 and enzyme (250 pM hFEN1 or 2000 pM R104AK132A), under identical buffer conditions to the ST reactions, as reported previously (3, 7). Triplicate reactions were initiated in 180 μl total volume, then 20 μl aliquots were quenched into 250 mM EDTA pH 8.0 at 2, 4, 6, 8, 10, 12 and 20 min (S1) or 8, 12, 16, 20, 25, 32 and 40 min (S8). Rate values were derived by linear regression in Microsoft Excel.

### Trapping and blocking experiments

Trapping/blocking experiments were conducted with substrate S2, bearing both carboxyfluorescein and biotin labels at the 5′-terminus of the 5′-flap, under saturating ST conditions using rapid quench-flow and manual sampling techniques as above. In each experimental run, ‘trapped’ reactions were conducted by adding streptavidin after incubation of substrate with the requisite hFEN1 protein, whereas streptavidin was mixed with the substrate before incubation with the hFEN1 protein for ‘blocked’ experiments, as detailed in earlier reports (7, 8). Control reactions were conducted as for the trapped reactions, except blank buffer was added instead of streptavidin. For all reactions, 8 M urea containing 300 mM EDTA (pH 8.0) was used as the quench solution. Quenched reaction aliquots were analyzed by HPLC as described above, then data were processed in GraphPad Prism 7 or later as documented previously (7).

### Fluorescence spectroscopy

Fluorescence measurements were conducted using substrate S3, which contains adjacent 2AP nucleobases at the +1 and −1 positions relative to the scissile phosphate of the reacting DNA strand (see Fig. 1*A*). Assays used 2.5 μM of wt hFEN1 or the D34N mutant and 0.5 μM of S3, mixed with 1 mM EDTA to produce a pre-formed complex as described (17). To initiate the experiment, 8 mM CaCl_2_ or MgCl_2_ was added as appropriate, then reactions were monitored at various time points from 5 to 100 s using a FluoroMax-3 instrument (Horiba Ltd) at 37 °C with a slit length of 5 nm. Excitation and emission wavelengths were 315 nm and 375 nm, respectively. Raw fluorescence values were normalized relative to reference values (*i.e.*, readings for samples with EDTA only, where no metal ions were added).

### ECCD assay

Measurements of the ECCD signal produced by two tandem 2AP residues in the indicated substrates (S4–S7 and S9; Fig. S1) were conducted as described (7). Briefly, spectra were acquired from samples containing 10 μM DNA and 12.5 μM protein in buffer CaRB (50 mM HEPES pH 7.5, 100 mM KCl, 10 mM CaCl_2_; 500 μl total sample volume) at 20 °C, using a Chirascan Plus CD spectrometer (Applied Photophysics Ltd). Processing of raw data relied on the Pro-Data Viewer 4.4.2.0 software supplied with the instrument. Spectra were baseline-corrected by subtracting a blank (recorded with CaRB) then subjected to smoothing (window size = 10), then the data were transformed to units of Δε per mol 2AP residue. Peak heights at 326 nm (downstream substrates) or 330 nm (upstream substrate) were used to quantify the extent to which different proteins are able to induce DNA conformational change. Due to variation in signal amplitude between substrate batches, values for each replicate were calculated relative to a substrate-only sample recorded on the same experimental run. Representative plots (Figs. 4*A* and 5, *E* and *G*) were prepared in GraphPad Prism 10.

### Sequence analysis

Sequence analysis was conducted as documented previously (7). To summarize, reviewed FEN1 sequences (archaea, n = 51; eukaryotes, n = 128) from the UniProt database were analyzed *via* multiple sequence alignment with ClustalX 2.1 (28), then the region corresponding to hFEN1 residues 86 to 133 was extracted and realigned. The output was used to generate sequence logos (Fig. 2*A*) with WebLogo 3 (29).

### Statistical analysis

Pairwise comparisons in Figures 5 and 6 used two-tailed, two-sample, unequal variance Student’s t-tests in Microsoft Excel. Statistical significance is denoted by asterisk(s) according to the following limits: *p* < 0.05 (∗), <0.01 (∗∗), <0.001 (∗∗∗), <0.0001 (∗∗∗∗). All datasets in Figures 4, *B*–*D*, 5, *F* and *H* were analyzed *via* Shapiro–Wilk Test in GraphPad Prism 11, and the assumption of normality was met in every case.

## Data availability

All data are contained within the manuscript and supporting information. Raw data supporting the results of this study will be provided by the corresponding authors upon request.

## Supporting information

This article contains supporting information.

## Conflict of interest

The authors have no conflicts of interest to declare regarding the content of this article.
