# Supplementary material for: Functional roles for FEN1 phosphate steering residues in multi-step substrate verification prior to reaction
Source: J Biol Chem. 2026 Jun 4;302(7):113225. doi: 10.1016/j.jbc.2026.113225 (PMC13333337; doi:10.1016/j.jbc.2026.113225)
Supplement: Supporting Information [file mmc1.pdf]

Supporting Information

Functional roles for FEN1 phosphate steering residues in allosterically controlled multi-step substrate verification leading to reaction

Mark J. Thompson, Nur Nazihah B. Md Shahari, Reuben J. Ouanounou, Nathan Gittens, Barbara Ciani, L. David Finger, and Jane A. Grasby*

School of Mathematical and Physical Sciences, University of Sheffield, Sheffield, UK

Table S1. Sequence information for oligonucleotides and DNA substrates.

**Oligonucleotide Sequences**		
**Oligo Number**	**Sequence**	
1	5′-GTGTCGAGCAGTCCTTGTGACGACGAAGTCGTCC-3	
2	5′-(6-FAM)-TTTTTACAAGGACTGCTCGACAC-3′	
3	5′-GAGTCCCATCTGCCTTTCGACAGCGAAGCTGTCC-3′	
4	5′-Biotin-5′-(6-FAM)-TTTTTGAAAGGCAGATGGGACTC-3′	
5	5′-ATGGGACGTGCTGTCAGTTACTGTGACCGTCAGGATGACACGC-3′	
6	5′-GCGTGTCATCCTGACGGTCACAGTG-3′	
7	5′-GTCGATCAGCTACG(2AP)(2AP)CTGACAGCACGTCCCAT-3′	
8	5′-CACTCTGCCTTTCGACAGCGAAGCTGTCC-3′	
9	5′-TTTTTG(2AP)(2AP)AGGCAGAGTG-3′	
10	5′P-G(2AP)(2AP)AGGCAGAGTG-3	
11	5′-CACTCTGCCTCTTGACAGCGAAGCTGTCC-3′	
12	5′-TTTTT(2AP)(2AP)GAGGCAGAGTG-3′	
13	5′P-(2AP)(2AP)GAGGCAGAGTG-3′	
14	5′-(6-FAM)-TTTT(dTMe)ACAAGGACTGCTCGACAC-3′	
15	5′-TTCGAGAAAGGCAGAGTG-3′	
16	5′-CACTCTGCCTTTCTTCAGCGAAGCTG(2AP)(2AP)-3′	
**Substrate Constructs**		
**Name**	**Oligonucleotides Used**	**Molar Ratio**
S1	1 + 2	1.1 : 1
S2	3 + 4	1.1 : 1
S3	5 + 6 + 7	1.1 : 1.1 : 1
S4	8 + 9	1.1 : 1
S5	8 + 10	1.1 : 1
S6	11 + 12	1.1 : 1
S7	11 + 13	1.1 : 1
S8	1 + 14	1.1 : 1
S9	15 + 16	1.1 : 1

Note: 6-FAM = 6-carboxyfluorescein, 2AP = 2-aminopurine, dTMe = 2′-deoxythymidine methylphosphonate.

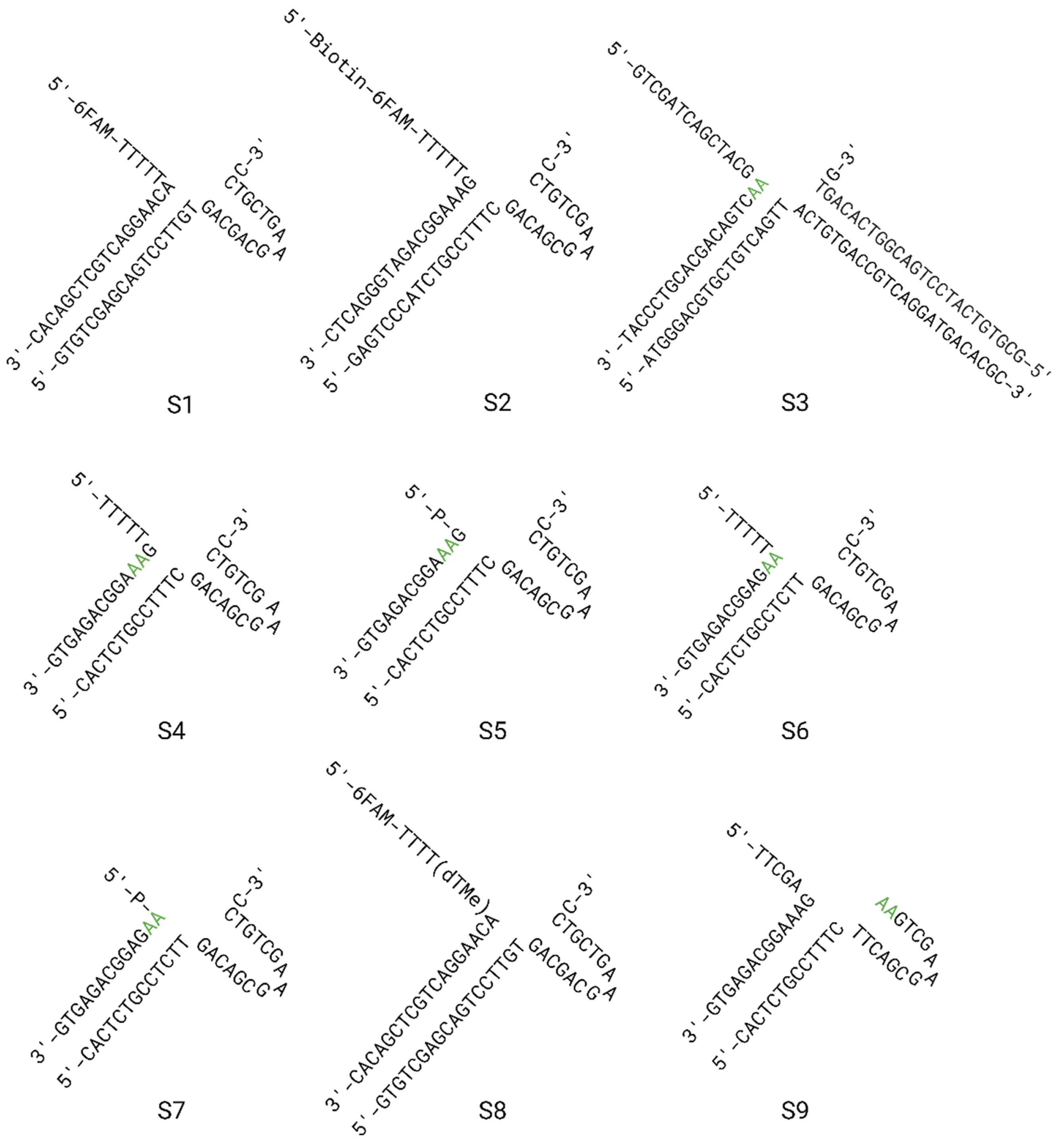

Figure S1. Structural schematics of DNA substrates S1–S9. The 6-FAM label in substrates S1, S2 and S8 was introduced using 5′-fluorescein-CE-phosphoramidite, while in S3, 5′-terminus of oligonucleotide 4 was extended by 6-fluorescein serinol phosphoramidite and BiotinTEG phosphoramidite in sequence to incorporate both 5′-labels. In oligonucleotide 14, the deoxythymidine-3′-methylphosphonate moiety was introduced using 5′-dimethoxytrityl-2′-deoxythymidine, 3′-[(methyl)-(*N*,*N*-diisopropyl)]-phosphonamidite. In substrates S3–S7 and S9, green ‘A’ labels indicate 2-aminopurine nucleotides.

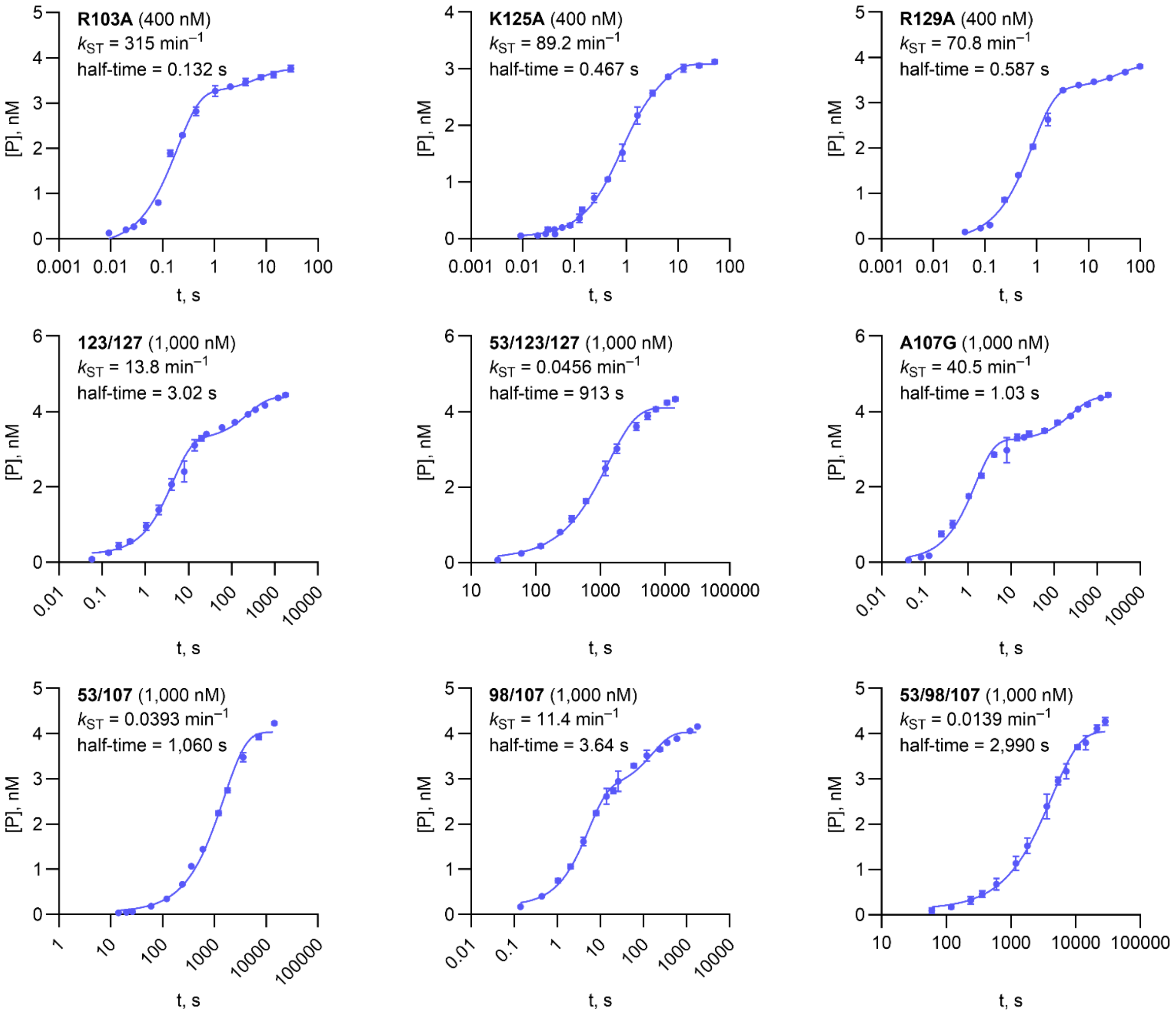

Figure S2. Single turnover reaction profiles with substrate S1 (5 nM) and the hFEN1 mutants indicated. Kinetic parameters (mean and standard error) were derived through model fitting in GraphPad Prism 10, using the two-phase association model (or one-phase association for 53/123/127, 53/107 and 53/98/107). Graphed points show the mean and SD of at least n = 3 independent replicates. Where time points of both <5 s and >100 s were included, independent experiments were conducted using the rapid quench flow method (QF) for shorter times and manual sampling for longer times. Replicates in each case were: R103A, n = 4 (QF) from two independent experiments performed in duplicate; K125A, n = 3 technical replicates (QF); R129A, n = 4 (QF) from two independent experiments performed in duplicate; 123/127, n = 4 (QF) from two independent experiments performed in duplicate plus n = 3 technical replicates (manual sampling for longer time points); 53/123/127, n = 6 from two independent experiments performed in triplicate (manual sampling); A107G, n = 4 (QF) from two independent experiments performed in duplicate plus n = 3 technical replicates (manual sampling for longer time points); 53/107, n = 3 technical replicates (manual sampling); 98/107, n = 4 (QF) from two independent experiments performed in duplicate plus n = 3 technical replicates (manual sampling for longer time points); 53/98/107, n = 6 from two independent experiments performed in triplicate (manual sampling).

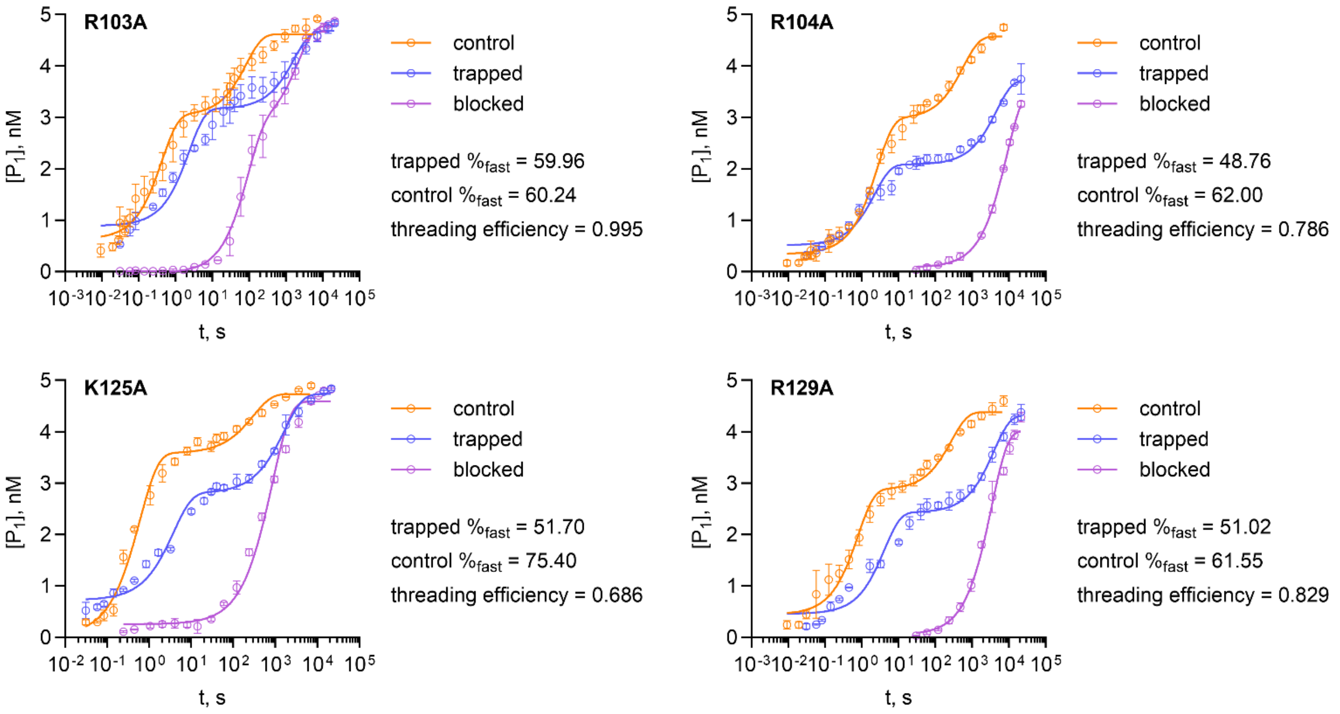

Figure S3. Trapped/blocked reaction profiles with substrate S2 (5 nM) for R103A, R104A, K125A and R129A (1 µM). Additional time course plots in the format shown in Fig. 3A, for the hFEN1 mutants indicated and used to determine threading efficiency for these proteins (Fig. 3B). Data points are plotted as the mean and SD of n = 4 replicates from two independent experiments performed in duplicate. The two-phase association model was used for curve fitting in GraphPad Prism 10, except for blocked reactions with R104A, K125A and R129A, where the single-phase association model was employed.

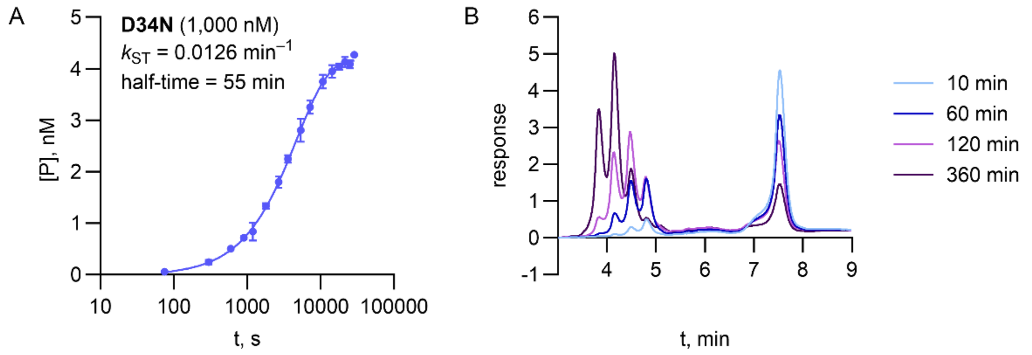

Figure S4. Single turnover kinetics results with D34N. *A*, Time course profile of the reaction with S1, plotting mean and SD of n = 4 replicates from two independent experiments performed in duplicate. *B*, representative HPLC chromatograms of reaction aliquots quenched at the time points indicated, showing gradual disappearance of substrate (t_R = 7.5 min) and appearance of multiple products at lower retention times, indicating off-target hydrolysis and/or rebinding and further hydrolysis of initial products. As such, D34N is a severely catalytically impaired mutant (>50,000× slower than wt hFEN1) that lacks specificity for the target reaction site.

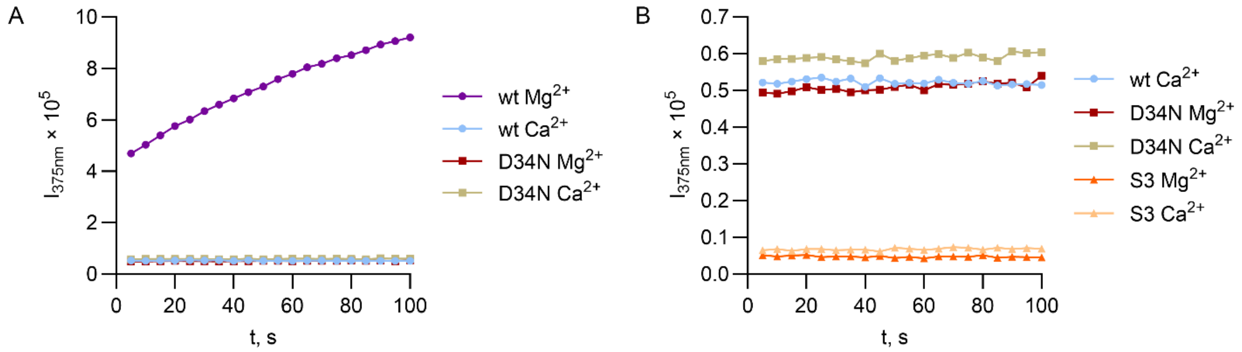

Figure S5. Fluorescence changes in hFEN1–S3 complexes with addition of catalytic (Mg^{2+}) or non-catalytic (Ca^{2+}) divalent metal ions. *A*, Relative 2AP fluorescence intensity at 375 nm (315 nm excitation) of 2AP-containing substrate S3 (0.5 μM) monitored from 5–100 s, in the presence of wt hFEN1 with 8 mM Mg^{2+} (purple plot) or Ca^{2+} (cyan plot), or hFEN1-D34N with 8 mM Mg^{2+} (red plot) or Ca^{2+} (beige plot). Protein concentration was 2.5 μM. *B*, Replot of samples producing no fluorescence change on a different y-axis scale, also including control fluorescence profiles for S3 with no added enzyme in the presence of 8 mM Mg^{2+} (orange plot) or Ca^{2+} (light orange plot). The results of a single experiment for each set of conditions are displayed.

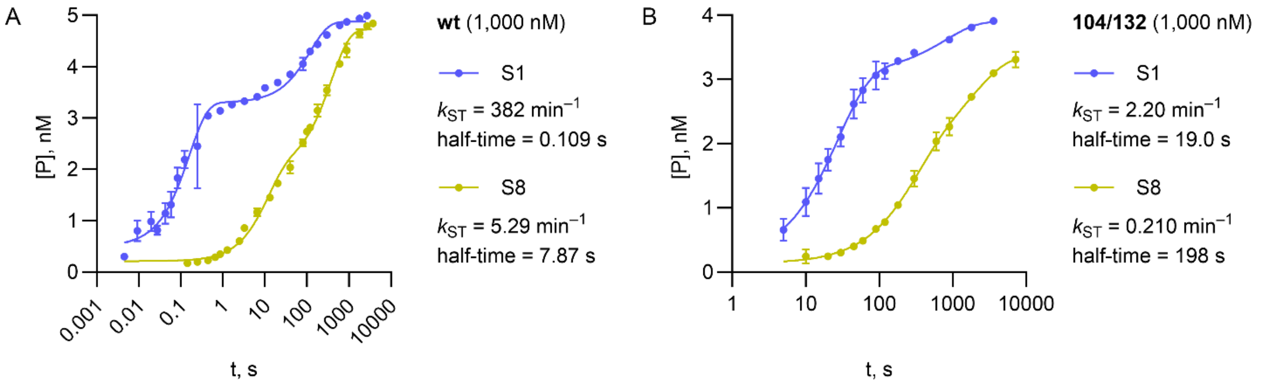

Figure S6. Reaction profiles with substrates S1 or S8 (5 nM), and wt hFEN1 or 'back-of-arch' double phosphate steering mutant 104/132 (1 μM). *A*, Time course profiles for S1 (blue plot) and S8 (beige plot) with wt hFEN1. Points are graphed as mean and SD of n = 3 technical replicates, and single turnover rate constants (k_{ST}) were derived via global fitting to the two-phase association model. *B*, Results for parallel experiments performed with 104/132. Points are also graphed as mean and SD of n = 3 technical replicates.
